# Radical prostatectomy technique in the robotic evolution: from da Vinci standard to single port—a single surgeon pathway

**DOI:** 10.1007/s11701-021-01194-8

**Published:** 2021-02-07

**Authors:** Simone Francavilla, Alessandro Veccia, Ryan W. Dobbs, Fabio Zattoni, Hari T. Vigneswaran, Alessandro Antonelli, Fabrizio Dal Moro, Riccardo Autorino, Claudio Simeone, Simone Crivellaro

**Affiliations:** 1 Department of Urology, College of Medicine, University of Illinois at Chicago, 820 S Wood Street, Chicago, IL 60612 USA; 2 Urology Unit, Department of Medical and Surgical Specialties, Radiological Science, and Public Health, ASST Spedali Civili Hospital, University of Brescia, Brescia, Italy; 3 Division of Urology, Department of Surgery, VCU Health System, Richmond, VA USA; 4 Urology Unit, Azienda Sanitaria Universitaria Integrata di Udine, Udine, Italy; 5 Urology Unit Azienda Ospedaliero Universitaria Integrata di Verona, Department of Surgery, Dentistry, Pediatrics and Gynecology, University of Verona, Verona, Italy

**Keywords:** Prostate cancer, Robotic surgery, Robot-assisted radical prostatectomy, Platforms comparison, Single port, Multiport

## Abstract

To describe perioperative outcomes following robot-assisted prostatectomy performed by a single surgeon during transitions between da Vinci standard/Si/Xi and the single port. Perioperative data were retrospectively evaluated of the first 40 consecutive robot-assisted radical prostatectomies performed by a single surgeon using the da Vinci standard, Si, Xi and single port. A total of 160 patients were included. We matched standard vs Si (Match 1), Si vs Xi (Match 2) and Xi vs single port (Match 3) cohort. Mann–Whitney and Fisher’s tests were used to test the difference among the groups. Univariate and multivariate logistic regression analyses were adopted to evaluate the predictors of overall and major complications. Single-port procedures in Match 3 showed significant shorter median operative time than Xi. Both Si and single-port groups showed significantly less median blood loss, a shorter median length of stay, respectively, than standard group in Match 1 and than Xi group in Match 3. 1 standard group patient required conversion to open surgery for an unsolvable conflict of the robotic arms. No other intraoperative complications were noted. On univariate and multivariate analyses, the da Vinci platform model was not a predicting factor of major complications (Clavien–Dindo ≥ 3). We described how technological progress impacted peri and postoperative outcomes during transitions between robotic surgical platforms for radical prostatectomy. In particular, the technological improvements associated to the increased surgeon’s expertise made the transition to the single port safe and effective when compared with previous platforms.

**Supplementary Information:**

The online version contains supplementary material available at 10.1007/s11701-021-01194-8.

## Introduction

Since its initial approval in 2000 by the United States Food and Drug Administration (FDA), the da Vinci surgical robot (Intuitive Surgical Inc. Sunnyvale, CA) has been rapidly adopted by urologists for performing radical prostatectomy (RP) [1]. Between 2003 and 2013, the proportion of Robot-Assisted Radical Prostatectomy (RARP) rose from 1.8 to 85%[2] and since the release of the initial da Vinci standard (S) model, there have been several iterations of this platform released including the Si (2009), Xi (2014) and single-port (SP) (2018) systems.

Transitions in surgical technology represent both a possible technological improvement as well as the potential to impact patient safety. While marketing for these technologies often focus on novel functionalities or features, if surgeons are unfamiliar or inexperienced with these systems, there is a potential for adverse patient outcomes. In particular, surgical robotics have been criticized for a reliance on unproven marketing claims [3] as well as evidence suggesting an increased prevalence of adverse patient safety events for RP early in the diffusion period of this technology [4]. As such, it is critically important to assess operative outcomes to ensure that transitions in surgical technology are not associated with increased risks for patients. While prior surgical robots adopted a similar configuration utilizing multiple ports and rigid laparoscopic instruments, the SP system represents a more significant departure from prior multiport (MP) platforms with a single robotic trocar and flexible articulating camera and instruments [5]. Early investigations have demonstrated the utility of the SP platform across a variety of surgical operations including prostatectomy [6–9], partial nephrectomy [10], pyeloplasty [11], vaginoplasty [12] and ureteral re-implantation [13].

To date, only one single surgeon multi-platforms comparison study in urology has been described which was for robot-assisted radical nephroureterectomy [14]. Currently, no investigator has reported their outcomes during transitions across multiple robotic platforms to examine how the introduction of a new platform impacts perioperative and pathological outcomes of interest for RARP.

The aim of this study was to describe the approach to each new platform comparing the outcomes among the first 40 RARP performed by a single surgeon using da Vinci S, Si, Xi & SP platform, evaluating if the transition to novel robotic technology was associated with adverse patient outcomes.

## Materials and methods

### Patient cohort

After Institutional Review Board approval at all participating Institutions, we retrospectively collected data of the first 40 consecutive RARPs performed by a single surgeon using the Da Vinci S, Si, Xi and SP platforms. Overall, 160 patients were included and classified into four groups according to the robot used. No specific exclusion criteria were used beyond case order as previously described. A 40-case cut-off was selected a priori according to literature regarding the multi-platforms comparison study [14]

The first 40 consecutive RARP were performed using the da Vinci standard between December 2009 and May 2011 at the University of Udine, Italy. These were the first cases performed by the surgeon as an attending following completion of a 1-year minimally invasive urology fellowship.

The da Vinci Si cohort consisted of the first 40 consecutive RARP performed between May 2014 and October 2015 at the University of Illinois at Chicago (UIC). At this point, the surgeon had an approximately 200 RARPs experience with the da Vinci S platform.

The da Vinci Xi cohort included the first 40 consecutive RARP performed between February 2016 and December 2016 at UIC. Overall, the surgeon had an experience of 270 RARPs with the S and Si platforms.

The da Vinci SP cohort included 40 consecutive RARP performed between December 2018 and October 2019 at UIC. Prior to these operations, the attending surgeon had performed approximately 400 multiport RARPs.

### Surgical technique

The RARP surgical technique adopted for S, Si and Xi has been previously described [15] and the anastomosis was performed with a double‐armed running suture according to Van Velthoven technique [16]. SP-RARPs were performed with a few technical changes from the MP-RARP technique as previously described [6].

### Clinical variables

Preoperative, perioperative, and postoperative parameters were evaluated according to STROBE protocol [17]. Complications were assessed according to the Clavien–Dindo classification (major: Clavien ≥ 3) [18]. All specimens were reviewed by a specialist uropathologist. Positive surgical margins (PSM) were defined as the presence of tumor at the inked surface of the specimen. Continence was defined as 0 or 1 (safety) pad per day.

### Statistical analysis

Statistical analysis followed the guidelines for reporting of statistics for clinical research in urology [19]. We focused the analysis on consecutive systems to reduce the bias associated with the increasing surgeon’s expertise across cohorts. For this reason, matched the da Vinci S cohort to the da Vinci Si cohort (Match 1), the da Vinci Si cohort to the da Vinci Xi cohort (Match 2) and finally the da Vinci Xi cohort to the da Vinci SP (Match 3). Continuous variables were reported as median [interquartile range (IQR)], whereas proportions and percentages were adopted for dichotomous data. The Mann–Whitney *U* test was deemed as appropriate to assess the difference among continuous variables. The Fisher’s exact test was used to evaluate the difference between dichotomous variables. Univariate and multivariate logistic regression analyses were used to evaluate the predictors of overall and major complications and included age at the surgery, D’Amico risk group, clinical and pathological International Society of Urological Pathology (cISUP and pISUP) group, and robotic platform model as covariates. All analyses were performed with Stata® 15.0 (StataCorp 2017. Stata Statistical Software: release 15. StataCorp LLC, College Station, TX, USA), and statistical significance was set at *p* < 0.05. The statistical codes adopted were: *ranksum, tabulate exact, logistic.*

## Results

### Preoperative variables

Table 1 shows preoperative and population features. Patients in the S group were significantly older [67.5 (61.0–70.5) vs 59.0 (58.0–64.5) years; *p* < 0.01] and showed lower preoperative Prostate Specific Antigen (PSA) [4.9 (3.7–6.4) vs 7.0 (5.6–12.0) ml/dl; *p* < 0.01] compared to the Si group in the match 1. Clinical ISUP was significant inferior in the S group compared to the Si group: 1/40 vs 22/40 patients with high-risk disease (cISUP > 2) [*p* < 0.01]. While BMI increased progressively, these differences were not statistically significant between the groups in any of the matches. The D’Amico risk groups were significantly higher in the Si group compared to S group in match 1 (*p* < 0.01) and in the Xi group compared to the SP group in Match 3 (*p* < 0.01).

**Table 1:** Preoperative and population features

	Match 1			Match 2			Match 3		
	Standard (*n* = 40)	Si (*n* = 40)	*p*	Si (*n* = 40)	Xi (*n* = 40)	*p*	Xi (*n* = 40)	SP (*n* = 40)	*p*
Median (IQR) age (years)	67.5 (61.0–70.5)	59.0 (58.0–64.5)	** < 0.01**	59.0 (58.0–64.5)	63 (57.0–66.5)	0.42	63 (57.0–66.5)	61.5 (58.0–65.0)	0.36
Median BMI (IQR) (kg/m^2^)	25.8 (24.2–28.0)	28.2 (23.3–30.7)	0.22	28.2 (23.3–30.7)	28.5 (25.4–32.4)	0.12	28.5 (25.4–32.4)	29.6 (25.4–30.5)	0.87
Median (IQR) Charlson comorbidity index	2 (1–2)	2 (1–3)	0.75	2 (1–3)	3 (2–3)	0.48	3 (2–3)	3 (2–4)	0.05
Median (IQR) preoperative PSA (ng/ml)	4.9 (3.7–6.4)	7.0 (5.6–12.0)	** < 0.01**	7.0 (5.6–12.0)	9.2 (5.0–15.5)	0.90	9.2 (5.0–15.5)	7.1 (6.5–12.1)	0.98
Biopsy grade group (ISUP)									
1	33 (83%)	9 (22.5%)	** < 0.01**	9 (22.5%)	9 (22.5%)	0.11	9 (22,5%)	6 (15.0%)	0.43
2	6 (15%)	9 (22.5%)		9 (22.5%)	16 (40.0%)		16 (40%)	22 (55.0%)	
3	1 (3%)	9 (22.5%)		9 (22.5%)	8 (20.0%)		8 (20%)	4 (12.5%)	
4–5	0 (0%)	13 (32.5%)		13 (32.5%)	7 (17.5%)		7 (17.5%)	8 (20.0%)	
Prostate cancer risk group (D’Amico)									
Low risk	31/40 (77.5%)	7/40 (17.5%)		7/40 (17.5%)	5 (12.5%)		5 (12.5%)	15 (37.5%)	
Intermediate risk	7/40 (17.5%)	18/40 (45.0%)	** < 0.01**	18/40 (45.0%)	22 (55%)	0.71	22 (55%)	14 (35.0%)	**0.03**
High risk	2/40 (5.0%)	15/40 (37.5%)		15/40 (37.5%)	13 (32.5%)		3 (32.5%)	11 (27.5%)	

Highlighted bold values are *p* values < 0.05

### Perioperative data

Intraoperative outcomes are described in Table 2. Si group showed significantly inferior blood loss 100 (50–150) vs 300 (150–300) ml [*p* < 0.01], a shorter length of stay 2 (2–3) vs 3 (3–4) days [*p* < 0.01], a shorter median postoperative urethral catheterization 9.0 (7.0–13.0) vs 10.0 (9.8–12.3) [*p* < 0.01] days compared to the standard group in Match 1.

**Table 2:** Intraoperative outcomes

	Match 1			Match 2			Match 3		
	Standard (*n* = 40)	Si (*n* = 40)	*p*	Si (*n* = 40)	Xi (*n* = 40)	*p*	Xi (*n* = 40)	SP (*n* = 40)	*p*
Median operative time (IQR) (Min)	270.0 (220.0–300.0)	262.5 (241.8–303.5)	0.71	262.5 (241.8–303.5)	270.5 (253.8–293.3)	0.60	270.5 (253.8–293.3)	238.5 (219.3–258.0)	**< 0.01**
Median estimated blood loss (IQR) (mL)	300 (150–300)	100 (50–150)	**< 0.01**	100 (50–150)	88 (50–100)	0.10	88 (50–100)	75 (50–125)	**< 0.01**
Median (IQR) length of stay (Day)	3 (3–4)	2 (2–3)	**< 0.01**	2 (2–3)	2 (2–3)	0.35	2 (2–3)	1 (1–1)	**< 0.01**
Median (IQR) postoperative urethral catheterization (Day)	10 (10–12)	10 (10–12)	**< 0.01**	9 (7–13)	9 (9–11)	0.14	9 (9–11)	9 (7–10)	**0.02**

Highlighted bold values are *p* values < 0.05

SP procedures in Match 3 showed significantly shorter operative times 238.5 (219.3–258.0) vs 270.5 (253.8–293.3) minutes [*p* < 0.01], and small but significantly reduced blood loss [75 (50–125) vs 88 (50–100) ml; *p* < 0.01], shorter median length of stay 1 (1–1) vs 2 (2–3) days [*p* < 0.01], and shorter median postoperative urethral catheterization 8.5 (7.0–10.0) vs 9.0 (9.0–11.0) [*p* 0.02] days as compared to the Xi group.

**Table 3:** Postoperative outcomes

	Match 1			Match 2			Match 3		
	Standard (*n* = 40)	Si (*n* = 40)	*p*	Si (*n* = 40)	Xi (*n* = 40)	*p*	Xi (*n* = 40)	SP (*n* = 40)	*p*
Pathological grade group (ISUP									
1	19/40 (47.5%)	2/40 (5%)	** < 0.01**	2/40 (5%)	3/40 (7.5%)	** < 0.01**	3/40 (7.5%)	1/40 (2.5%)	0.70
2	15/40 (37.5%)	11/40 (27.5%)		11/40 (27.5%)	23/40 (57.5%)		23/40 (57.5%)	24/40 (60.0%)	
3	2/40 (5%)	14/40 (35%)		14/40 (35%)	7/40 (17.5%)		7/40 (17.5%)	9/40 (22.5%)	
4–5	4/40 (10%)	13/40 (32.5%)		13/40 (32.5%)	7/40 (17.5%)		7/40 (17.5%)	6/40 (15.0%)	
Pathological stage									
pT2	32/40 (80%)	27/40 (67.5%)	0.47	27/40 (67.5%)	22/40 (55%)	0.58	22/40 (55%)	24/40 (60.0%)	0.82
pT3a	6/40 (15%)	9/40 (22.5%)		9/40 (22.5%)	12/40 (30%)		12/40 (30%)	9/40 (22.5%)	
pT3b/pT4	2/40 (5%)	4/40 (10%)		4/40 (10%)	6/40 (15%)		6/40 (15%)	7/40 (17.5%)	
pNx	36/40 (90%)	21/40 (52.5%)		21/40 (52.5%)	17/40 (42.5%)		17/40 (42.5%)	21/40 (52.5%)	
pN0	4/40 (10%)	17/40 (42.5%)	**< 0.01**	17/40 (42.5%)	19/40 (47.5%)	0.59	19/40 (47.5%)	11/40 (27.5%)	0.43
pN1	0 (0%)	2/40 (5%)		2/40 (5%)	4/40 (10%)		4/40 (10%)	6/40 (15.0%)	
Median lymph node yield (IQR)	5.0 (3.0–6.0)	7.5 (4.0–11.0)	0.34	7.5 (4.0–11.0)	9.0 (7.5–16.0)	**0.03**	9.0 (7.5–16.0)	12.0 (9.0–12.0)	0.88
Positive surgical margins	8/40 (20%)	11/40 (27.5%)	0.60	11/40 (27.5%)	21/40 (52.5%)	**0.04**	21/40 (52.5%)	15/40 (38.5%)	0.26
Major complications (CL-D > 3)	6/40 (15%)	5/40 (12.5%)	0.61	5/40 (12.5%)	5/40 (12.5%)	1.00	5/40 (12.5%)	5/40 (12.5%)	1.00
90-day undetectable PSA rate	37/40 (92.5%)	34/40 (85.0%)	0.74	34/40 (85.0%)	36/40 (90.0%)	0.74	36/40 (90.0%)	33/38 (86.8%)	0.73
90-day continence rate (0–1 pad/day)	29/39 (74.4%)	24/33 (72.7%)	0.48	24/33 (72.7%)	27/36 (75.0%)	1.00	27/36 (75.0%)	25/37 (67.6%)	0.61

Highlighted bold values are *p* values < 0.05

One standard group patient required conversion to an open surgical technique for an unsolvable conflict of the robotic arms. No additional intraoperative complications were noted.

### Postoperative data

Table 3 shows postoperative outcomes. Tumor aggressiveness (pISUP) was significantly lower in S group than Si in Match 1 (*p* < 0.01), in Xi group than Si group in Match 2 (*p* < 0.01) and in Xi than SP group in Match 3 (*p* < 0.01). S group showed inferior pT stage (*p* < 0.01) and a lower rate of lymphadenectomy compared to Si (*p* < 0.01). PSM were significantly higher in Xi than Si group in match 2 21/40 (52.5%) vs 11/40 (27.5%) [*p* 0.04].

Median length of stay and duration of postoperative catheterization were significantly shorter in Si group than Standard group in match 1 (*p* < 0.01) and in SP group than Xi group in Match 3 (*p* < 0.01). While major complication rates were similar between groups in all the matches.

The 90-day undetectable PSA rate and the 90-day continence rate were not significantly different in all the matches.

On univariate and multivariate analyses, the da Vinci platform model was not an independent predictor of major complications (Clavien–Dindo ≥ 3).

## Discussion

In this project, we sought to describe early, peri and postoperative outcomes for RARP during the transitional phase of a surgeon’s first 40 cases across 4 consecutive da Vinci platforms (standard, Si, Xi, and single port) to determine if transitions in technology were associated with adverse clinical outcomes. We focused the analysis on passage between consecutive robotic systems without comparing in a binary analysis multiport vs single port to reduce the bias associated with the increasing surgeon’s expertise across cohorts. For this reason, we matched the da Vinci S cohort to the da Vinci Si cohort (Match 1), the da Vinci Si cohort to the da Vinci Xi cohort (Match 2) and finally the da Vinci Xi cohort to the da Vinci SP (Match 3). We found that transitions in surgical technology were not a predictor of significant complications on either univariate or multivariate analysis; however, we did find improvements in estimated blood loss, median length of stay, and median urethral catheterization in Match 1 (the transition from the standard to the Si system) as well as Match 3 (the transition from the Xi to the SP system) and shorter operative times for the SP system as compared to its multiport predecessor.

This is the first experience of this genre reported in literature for RARP.

Previously, only few authors have assessed the impact of robotic platforms on patients’ outcomes. Patel et al. showed shorter operative time for the da Vinci Xi than the older da Vinci S/Si platforms during nephroureterectomy [14]. However, this difference was explained as resulting from to the lack of patient repositioning for the ureteral dissection using the Xi system. Other comparisons for partial nephrectomy [20], adrenalectomy [21] and prostatectomy [22] demonstrated that the Xi system provided shorter docking and console time as compared to the Si system. [20–22] Given that the SP platform is a new technology, no similar studies have been reported using this system.

As with many such studies, we found statistical differences between arms that can be explained though they may have occurred by chance and others that may have occurred by chance or are simply not as easily explained.

Within these series, the Match 1 (S vs Si) is affected by two bias. First, the surgeon’s lack of robotic experience before performing the S series (that were his first cases as an attending following a 1-year minimally invasive urology fellowship) could affect the differences in intra and postoperative outcomes with the Si series (performed by the surgeon after 200 prostatectomies). As such, the improvement in intraoperative outcomes between the S and Si operations may be resulted from increased surgeon experience and the primary surgeon’s learning curve, as described in literature [23, 24].

Additionally, the S group was performed in Italy and has significantly differences in preoperative and disease features from the Si population (which was mainly African American) performed in the United States; so differences in patient populations, combined with a movement toward active surveillance may contribute to the differences in pathology observed between groups. [25]

The Match 2 (Si vs Xi), showed only one difference in terms of PSM which were higher in the Xi cohort. An explanation might reside in the higher rate of pT ≥ 2 tumors and intrafascial nerve sparing procedures. This finding is not consistent with previous evidence [22]. The increased nodal yields are probably correlated with the increased surgeon experience [26]

The Match 3 (Xi vs SP) was chronologically placed in the plateau phase of the surgeon’s learning curve, with over 270 procedures. Since the learning curve for prostate cancer surgery, as measured by cancer recurrence, plateaus after approximately 250 operations [27], this may represent an advantage of the da Vinci SP to improve operative time as a result of simplified docking and undocking. Prior comparative studies demonstrated a similar improvement of approximately 20 min in the SP arm as compared to the Xi (255.9 ± 44.1 versus 274.7 ± 50.4 min, respectively), (*p* = 0.06) to the 32-min improvement seen in our experience (*p* < 0.01). Additionally, this comparison demonstrated small, but significant reductions in hospital stay resulting from improved patient recovery and convalescence which may be justified by the significantly pain-free rate in patients operated with the SP platform, as shown by Vigneswaran et al. analyzing the differences in robotic surgery for localized prostate cancer using a single-port robotic platform compared to the traditional multiport robotic platform [28].

This project represents a unique dataset as few surgeons have had the opportunity to perform a longitudinal series of RARPs across four different da Vinci models allowing to describe what happened in terms of outcomes during transitions in technology from the standard to the Si, from the Si to the Xi and from the Xi to the SP robotic system. An inherent confounding factor in our analysis is greater surgeon experience across each cohort, a bias that makes direct comparison of the four robots somewhat more challenging. To account for this, we focused on individual transitions from each platform to its successor. These findings also showed that the learning curve for SP-RALP is relatively short for experienced robotic surgeons, even without a previous single site surgical experience.

The main limitation of this study was the retrospective nature of the analysis and the non-randomization of the patients between groups. Although, the sample sizes were not large, this is a preliminary study and describes our preliminary perioperative outcomes with both the da Vinci standard, Si, Xi and SP robotic platforms. Another limitation was the 90-day follow-up period, not enough to evaluate oncological and functional outcomes, such as long-term biochemical recurrence and continence. Additional longitudinal observation is still required to evaluate the long-term outcomes. Despite these weaknesses, this represents a unique historical dataset that captures three critical moments of transition across four different robotic platforms.

Cost considerations are currently a valid concern for the acquisition of the da Vinci SP. Our institution had to afford an entirely new investment as there was no shared component with the Xi System, but the next generation may allow for compatibility for the console and with the Xi platform. Radical prostatectomy is one of the procedures that would benefit meaningfully from this technology, but the regionalization of care in designated reference centers may be necessary to evaluate a cost-effective analysis of the SP system.

## Conclusion

We described how technological transitions impacted peri and postoperative outcomes during transitions between robotic surgical platforms for RARP. In particular, the technological progress associated to the increased surgeon’s expertise made the transition to the da Vinci SP robotic system safe and effective when compared with previous platforms even in learning curve, showing shorter operative times, reduced blood loss, shorter length of hospitalization and shorter duration of urethral catheterization in favor of the SP system compared to the Xi.

## Supplementary Information

Below is the link to the electronic supplementary material.

Supplementary file1 (MP4 179599 KB)

## Data Availability

The data that support the findings of this study are available from the corresponding author, [SF], upon reasonable request.

## References

[1] Dobbs RW, Magnan BP, Abhyankar N (2017). Cost effectiveness and robot-assisted urologic surgery: does it make dollars and sense?. Minerva Urol Nefrol.

[2] Leow JJ, Chang SL, Meyer CP (2016). Robot-assisted versus open radical prostatectomy: a contemporary analysis of an all-payer discharge database. Eur Urol.

[3] Schiavone MB, Kuo EC, Naumann RW (2012). The commercialization of robotic surgery: unsubstantiated marketing of gynecologic surgery by hospitals. Am J Obstet Gynecol.

[4] Mirheydar HS, Parsons JK (2013). Diffusion of robotics into clinical practice in the United States: process, patient safety, learning curves, and the public health. World J Urol.

[5] Dobbs RW, Halgrimson WR, Talamini S, Vigneswaran HT, Wilson JO, Crivellaro S (2020) Single-port robotic surgery: the next generation of minimally invasive urology. World J Urol 38:897–905 10.1007/s00345-019-02898-1 31463560

[6] Dobbs RW, Halgrimson WR, Madueke I, Vigneswaran HT, Wilson JO, Crivellaro S (2019). Single-port robot-assisted laparoscopic radical prostatectomy: initial experience and technique with the da Vinci(I) SP platform. BJU Int.

[7] Kaouk J, Aminsharifi A, Wilson CA et al (2020) Extraperitoneal versus transperitoneal single-port robotic radical prostatectomy: a comparative analysis of perioperative outcomes. J Urol 203:1135–1140 10.1097/JU.0000000000000700 31846392

[8] Ng CF, Teoh JY, Chiu PK (2019). Robot-assisted single-port radical prostatectomy: a phase 1 clinical study. Int J Urol.

[9] Moschovas MC, Bhat S, Rogers T, Onol F, Roof S, Mazzone E, Mottrie A, Patel V (2020). Technical modifications necessary to implement the da vinci single-port robotic system. Eur Urol.

[10] Kaouk J, Garisto J, Eltemamy M, Bertolo R (2019). Pure single-site robot-assisted partial nephrectomy using the sp surgical system: initial clinical experience. Urology.

[11] Heo JE, Kang SK, Koh DH (2019). Pure single-site robot-assisted pyeloplasty with the da Vinci SP surgical system: Initial experience. Investig Clin Urol.

[12] Acar O, Sofer L, Dobbs RW et al (2020) Single port and multi-port approaches for robotic vaginoplasty with the davydov technique. Urology 2020 138:166–173 10.1016/j.urology.2019.11.043 31904396

[13] Hebert KJ, Joseph J, Gettman M, Tollefson M, Frank I, Viers BR (2019). Technical considerations of single port ureteroneocystostomy utilizing da Vinci SP platform. Urology.

[14] Patel MN, Hemal AK (2018). Does advancing technology improve outcomes? Comparison of the Da Vinci Standard/S/Si to the Xi robotic platforms during robotic nephroureterectomy. J Endourol.

[15] Patel VR, Coelho RF, Palmer KJ, Rocco B (2009). Periurethral suspension stitch during robot-assisted laparoscopic radical prostatectomy: description of the technique and continence outcomes. Eur Urol.

[16] Van Velthoven RF, Ahlering TE, Peltier A, Skarecky DW, Clayman RV (2003). Technique for laparoscopic running urethrovesical anastomosis:the single knot method. Urology.

[17] von Elm E, Altman DG, Egger M (2007). The strengthening the reporting of observational studies in epidemiology (STROBE) statement: guidelines for reporting observational studies. Lancet.

[18] Clavien PA, Barkun J, de Oliveira ML (2009). The Clavien-Dindo classification of surgical complications: five-year experience. Ann Surg.

[19] Assel M, Sjoberg D, Elders A (2019). Guidelines for reporting of statistics for clinical research in urology. J Urol.

[20] Abdel Raheem A, Sheikh A, Kim DK (2017). Da Vinci Xi and Si platforms have equivalent perioperative outcomes during robot-assisted partial nephrectomy: preliminary experience. J Robot Surg.

[21] Feng Z, Feng MP, Feng DP, Solorzano CC (2020) Robotic-assisted adrenalectomy using da Vinci Xi vs. Si: are there differences? J Robot Surg 14: 349–355 10.1007/s11701-019-00995-2 31273609

[22] Goonewardene SS, Cahill D (2017). The Da Vinci Xi and robotic radical prostatectomy-an evolution in learning and technique. J Robot Surg.

[23] Ou YC, Yang CR, Wang J (2010). Robotic-assisted laparoscopic radical prostatectomy: learning curve of first 100 cases. Int J Urol.

[24] Ahlering TE, Skarecky D, Lee D, Clayman RV (2003). Successful transfer of open surgical skills to a laparoscopic environment using a robotic interface: initial experience with laparoscopic system. J Urol.

[25] Fuletra GJ, Anastasiya K, Ramsey F (2018). African–American men with prostate cancer have larger tumor volume than Caucasian men despite no difference in serum prostate specific antigen. Can J Urol.

[26] van der Poel HG, de Blok W, Tillier C, van Muilekom E (2012). Robot-assisted laparoscopic prostatectomy: nodal dissection results during the first 440 cases by two surgeons. J Endourol.

[27] Ou YC, Yang CK, Chang KS (2014). The surgical learning curve for robotic-assisted laparoscopic radical prostatectomy: experience of a single surgeon with 500 cases in Taiwan, China. Asian J Androl.

[28] Vigneswaran HT, Schwarzman LS, Francavilla S (2020). A Comparison of perioperative outcomes between single-port and multiport robot-assisted laparoscopic prostatectomy. Eur Urol.

